# MMP-10 is Increased in Early Stage Diabetic Kidney Disease and can be Reduced by Renin-Angiotensin System Blockade

**DOI:** 10.1038/s41598-019-56856-3

**Published:** 2020-01-08

**Authors:** José María Mora-Gutiérrez, José Antonio Rodríguez, María A. Fernández-Seara, Josune Orbe, Francisco Javier Escalada, María José Soler, María Fernanda Slon Roblero, Marta Riera, José Antonio Páramo, Nuria Garcia-Fernandez

**Affiliations:** 1 Nephrology Department, Clínica Universidad de Navarra, Pamplona, Spain; 2 Laboratory of Atherothrombosis, Program of Cardiovascular Diseases, CIMA, University of Navarra, Pamplona, Spain; 3 Radiology Department, Clínica Universidad de Navarra, Pamplona, Spain; 4 Endocrinology and Nutrition Department, Clínica Universidad de Navarra, Pamplona, Spain; 5 Nephrology Department, Hospital Universitari Vall d’Hebron, Barcelona, Spain; 6 Nephrology Department, Hospital de Navarra, Pamplona, Spain; 7 Nephrology Department, Hospital del Mar, IMIM (Hospital del Mar Medical Research Institute), Barcelona, Spain; 8 CIBER Cardiovascular (CIBERCV), Instituto de Salud Carlos III, Madrid, Spain; 9 CIBER Fisiopatología de la Obesidad y Nutrición (CIBEROBN), Instituto de Salud Carlos III, Madrid, Spain; 10 IdiSNA, Navarra Institute for Health Research, Pamplona, Spain; 11 Red de Investigación Renal (REDINREN), Instituto de Salud Carlos III, Madrid, Spain

**Keywords:** Diabetes complications, Podocytes

## Abstract

Matrix metalloproteinases have been implicated in diabetic microvascular complications. However, little is known about the pathophysiological links between MMP-10 and the renin-angiotensin system (RAS) in diabetic kidney disease (DKD). We tested the hypothesis that MMP-10 may be up-regulated in early stage DKD, and could be down-regulated by angiotensin II receptor blockade (telmisartan). Serum MMP-10 and TIMP-1 levels were measured in 268 type 2 diabetic subjects and 111 controls. Furthermore, histological and molecular analyses were performed to evaluate the renal expression of *Mmp10* and *Timp1* in a murine model of early type 2 DKD (db/db) after telmisartan treatment. MMP-10 (473 ± 274 pg/ml vs. 332 ± 151; p = 0.02) and TIMP-1 (573 ± 296 ng/ml vs. 375 ± 317; p < 0.001) levels were significantly increased in diabetic patients as compared to controls. An early increase in MMP-10 and TIMP-1 was observed and a further progressive elevation was found as DKD progressed to end-stage renal disease. Diabetic mice had 4-fold greater glomerular *Mmp10* expression and significant albuminuria compared to wild-type, which was prevented by telmisartan. MMP-10 and TIMP-1 are increased from the early stages of type 2 diabetes. Prevention of MMP-10 upregulation observed in diabetic mice could be another protective mechanism of RAS blockade in DKD.

## Introduction

Diabetic kidney disease (DKD) is a leading cause of end-stage renal disease. Matrix metalloproteinases (MMPs) have been found to play a role in various pathogenic mechanisms involved in microvascular complications of diabetes mellitus (DM). These endopeptidases are implicated in the degradation and remodelling of the extracellular matrix (ECM), as well as proteolytic modification of growth factors and cytokines^1^. MMP activity is regulated by specific tissue inhibitors of matrix metalloproteinases (TIMPs).

Higher circulating MMP-2 and MMP-9 levels have been found in diabetes^2,3^. An association between high serum levels of MMP-10 and a greater risk of DKD has been previously shown in type 1 diabetes (T1DM), and the absence of *Mmp10* in a murine model protected against renal macrophage infiltration and mesangial expansion^4^. We and others have demonstrated that serum concentration of MMP-10 is elevated in chronic kidney disease (CKD) associated with vascular complications^5,6^. Previous studies on the endogenous inhibitor of MMP-10, TIMP-1, show inconclusive data, demonstrating elevated circulating levels on DM^5^, while others observed similar levels as compared to healthy subjects^7^.

The renin-angiotensin system (RAS) is crucial in the pathogenesis of DKD. Hyperglycaemia stimulates local RAS activation generating changes in podocytes and glomerular basement membrane thickness^8^. Moreover, RAS inhibition is one of the most effective therapies to delay renal disease progression in diabetes. Interestingly, previous reports have shown that RAS blockage inhibits MMP-2 activation in diabetic rats^9^ and, additionally, MMP-9 expression and activity, triggered by advanced glycation end products, was attenuated by olmesartan^10^. No data linking RAS activation and renal *Mmp10* expression has been previously reported.

MMP-10 and TIMP-1 have been implicated in T1DM as described above, however, to the best of our knowledge, no previous studies have analysed the role of MMP-10 in type 2 diabetes (T2DM), while TIMP-1 data is not conclusive. Our hypothesis is that MMP-10 may be up-regulated in early stage DKD, and could be down-regulated by angiotensin II receptor blockade (telmisartan). The clinical study aimed to assess circulating levels of MMP-10 and TIMP-1 in T2DM, in different stages of DKD. In addition, an experimental study was performed to analyse renal *Mmp10* and *Timp1* expression in a mouse model of early DKD, and their potential modulation by RAS blockade.

## Methods

### Subjects and samples

A total of 324 consecutive patients with type 2 diabetes mellitus, attending the Endocrinology Department at Clínica Universidad de Navarra (CUN, Pamplona, Spain) and Nephrology Departments at CUN and Hospital de Navarra (Pamplona, Spain), were recruited over a period of 24 months for the cross-sectional observational study. Of these, 11 declined participation in the study and 45 patients did not fulfil inclusion criteria (see Supplementary Fig. S1).

The study was approved by the Ethics Committee of University of Navarra and Hospital de Navarra in Pamplona, Spain. All procedures performed in this study were in accordance with the ethical standards of the institutional and/or national research committee and with the 1964 Helsinki declaration and its later amendments or comparable ethical standards. The study was approved by the University of Navarra Ethical Committee. Written informed consent was obtained from all subjects before inclusion.

The inclusion criteria were: diagnosis of type 2 diabetes mellitus at least 5 years before inclusion, >18 years of age and eGFR higher than 60 ml/min/1.73 m^2^ with albuminuria greater than 30 mg/g, or eGFR lower than 60 ml/min/1.73 m^2^ regardless of the albuminuria degree. Exclusion criteria included: immunosuppressive treatment, active autoimmune or neoplastic disease, or any possible aetiology of CKD other than diabetes. Healthy (normotensive, non-diabetic) subjects (n = 111), attending regular medical check-ups at CUN, with normal renal function and without RAS inhibitor treatment, were recruited as control group.

Clinical, analytical and demographic variables were collected from all subjects. Serum creatinine and cystatin C were determined by nephelometry on a BN Prospec autoanalyzer (Siemens, Erlangen, Germany). The GFR was estimated by Modification of Diet in Renal Disease-4 (MDRD-4) and Chronic Kidney Disease Epidemiology Collaboration (CKD-EPI) formulas, using serum creatinine and/or cystatin C. Glucose levels were measured using the AU5800 autoanalyzer (Beckman Coulter, Brea, CA, USA) and serum insulin with IMMULITE-2000 (Siemens, Erlangen, Germany). Urinary albumin/creatinine ratio (uACR) was measured in the sample spot. Subjects with diabetes were classified according to their GFR (ml/min/1.73 m^2^), estimated by CKD-EPI cystatin C, as group 1 (eGFR > 90), group 2 (eGFR 90–60), group 3 (eGFR 60–30) and group 4 (eGFR < 30); and according to their stage of albuminuria, as A1 (normoalbuminuria: <30 mg/g), A2 (microalbuminuria: 30–300 mg/g) and A3 (macroalbuminuria: >300 mg/g).

### Serum MMP-10 and TIMP-1 measurement

Serum levels of MMP-10 (DM1000, R&D Systems, Abingdon, UK; dilution 1:2) and TIMP-1 (DY970, R&D Systems, Abingdon, UK; dilution 1:100) were assessed by ELISA according to the manufacturer’s instructions. The detection limit for MMP-10 was 78.1 pg/ml and for TIMP-1 was 0.15 ng/ml. Inter- and intra-coefficients of variation were <8% in both analyses.

## Experimental methods

All procedures performed in studies involving animals were conducted according to the European Community guidelines for the ethical animal care and use of laboratory animals (2010/63/EU) and approved by the University of Navarra Animal Research Review Committee. Five weeks-old male db/db (BKS.Cg-+*Lepr*^*db*^*/*+*Lepr*^*db*^/OlaHsd) and db/m (BKS.Cg-+*Lepr*^*db*^/OlaHsd) mice were purchased from Envigo (Venray, Netherlands). Mice were maintained on a 12-hour light/12-hour dark cycle, had free access to water and were fed with standard chow diet (db/m) or 2018-chow diet (db/db) (Teklad Global 18%-Protein Rodent Diet, Envigo, Madison, WI, USA). Animals were divided into four groups (n = 6), db/db (diabetic); db/db telmisartan-treated (5 mg/kg/day starting at 8 weeks of age); db/m (non-diabetic); db/m telmisartan-treated. Two days before being sacrificed, blood pressure was measured using a system adapted to the mouse tail, following manufacturer’s instructions (Mouse/Rat Tail Cuff Blood Pressure System, IITC Life Science, Woodland Hills, CA, USA), and 24-hour urine was collected in metabolic cages. Mice were sacrificed by CO_2_ inhalation at 8, 12 and 16 weeks of age, and kidneys and blood were collected.

### Analytical measurements

Measurements of plasma creatinine and glucose concentrations, as well as urine creatinine and albumin, were performed using specific kits on a Cobas C311 biochemical analyser (Roche, Mannheim, Germany). Plasma insulin concentration was determined by ELISA (Merck-Millipore EZRMI-13K, Billerica, MA, USA).

### Kidney histological analysis

Sections (3-μm-thick) cut from 10% formalin-fixed, paraffin-embedded kidney samples were used for periodic acid–Schiff (PAS) and Masson trichrome staining. Using coronal sections of the kidney, 10–15 consecutive glomeruli per mouse (6 mice per group) were examined for evaluation of glomerular mesangial expansion. The index of the mesangial expansion was defined as the ratio of mesangial area to glomerular tuft area. The mesangial area was determined by assessment of the PAS positive and nucleus-free area in the mesangium using ImageJ software.

### Immunohistochemistry

MMP10 immunostaining was performed in renal tissue. Briefly, after citrate-induced antigen retrieval, endogenous peroxidase was blocked with 3% H_2_O_2_. Sections were incubated overnight at 4 °C with a polyclonal rabbit anti-human MMP10 antibody (1:20, AP07210PU-N, Acris-Antibodies, San Diego, USA) in 1% bovine serum albumin. Sections were then incubated with a specific detection system (EnVision+ System HRP-labelled polymer anti-rabbit, K4002, Dako, Glostrup, Denmark) for 30 min at room temperature. Peroxidase activity was revealed using 3,3’-diaminobenzidine-tetrahydrochloride (DAB+, K3468, Dako, Glostrup, Denmark), and sections were counterstained with Harris’ haematoxylin, dehydrated and mounted on DPX. Slides were analysed under the microscope in a blinded manner.

### Reverse transcription and Real-time PCR

Renal tissue RNA was extracted (n = 6 kidneys for each experimental group) with a semi-automated system (ABI-PRISM 6100, Applied-Biosystems, Warrington, UK), according to the manufacturer’s instructions, and reverse transcribed with Moloney Murine Leukaemia Virus reverse transcriptase (Invitrogen, Thermo Fisher Scientific, Carlsbad, CA, USA). qPCR was performed using the ABI-PRISM 7900HT thermal cycler (Applied-Biosystems, Warrington, UK) with a predesigned set of TaqMan primers and probes specific for *Mmp10* (Mm.PT.42.8739011-IDT) and *Timp1* (Mm.PT.58.30682575-IDT). *β-actin* (Mm.PT.49.9990212.g-IDT) was used as the housekeeping gene.

### Statistical analysis

Parametric tests were used for statistical analyses in the clinical study due to the large sample size. Data are expressed as means (SD), median (IQR) or proportions, as appropriate. Differences in demographic and clinical variables between the patient and control groups were evaluated using unpaired Student’s *t* tests for quantitative variables, and the Chi-Square tests for categorical variables. Since the groups were found to differ in age, gender frequency and BMI, group differences in MMP-10 and TIMP-1 were evaluated using a generalized linear model, including: group (patients with diabetes or healthy subjects), age, gender and BMI to control for the possible confounding effect. Associations between variables were examined using Pearson correlation. Changes in MMP-10 and TIMP-1 across different stages of CKD or albuminuria were evaluated using two way ANCOVA, introducing age, gender and BMI as co-variables of confusion. When appropriate, *post-hoc* contrasts of the adjusted means were performed using Tukey correction. For the experimental study, differences between groups of mice were analysed using ANOVA, followed by appropriate *post-hoc* tests. Associations between renal *Mmp10* expression and other variables were analysed by Pearson correlation, and adjusted per group by multiple linear regression. P*-*values were penalized by Bonferroni and statistical significance was established at p < 0.05. Statistical analysis was performed using R 3.0.3^11^.

## Results

### Clinical Study

#### Population characteristics

Table 1 shows the demographic and clinical characteristics of the participants. There was a higher proportion of males among patients with diabetes (38% vs. 73%), who were also older and had a higher BMI compared to controls. Prevalence of hypertension among diabetics was 77%, and 70% of them received RAS inhibitors. Diabetic subjects using RAS inhibitors had a greater albuminuria compared to those without RAS blockade (40.58 ± 40.58 vs 22.64 ± 74.62 mg/mmol, p = 0.009).

**Table 1:** Demographic and clinical characteristics of the study population.

	Controls n = 111	T2DM n = 268	*p-value*
**Gender**, female/male	69/42	73/195	<0.01
**Age**, years	56.9 (11.4)	66.7 (10.6)	<0.01
**BMI**, kg/m^2^	25.6 (3.9)	28.9 (4.5)	<0.001
**Duration of diabetes**, years	N/A	11.4 (8.7)	
**Diabetic retinopathy**, %	N/A	17.5	
Non-Proliferative		55.3	
Proliferative		44.7	
**Smoking**, (no/former/current) %	70/16/14	54/28/18	
**Hypertension**, %	0	76.5	<0.01
Systolic BP, mmHg	121 (15)	134 (19)	<0.001
Diastolic BP, mmHg	75 (10)	75 (9)	NS
**Vascular disease**, %	8.0	34.7	<0.05
**Serum cystatin C**, mg/l	8 (1.3)	12.6 (7.2)	<0.001
**Serum creatinine**, μmol/l	71.6 (15.03)	108.73 (76.02)	<0.001
**eGFR**, ml/min/1.73 m^2^	95.77 (14.77)	71.64 (30.87)	<0.001
**Albuminuria**, mg/mmol	0.83 (0.86)	38.54 (105.27)	<0.001
**FPG**, mmol/l	5.11 (0.72)	7.77 (2.61)	<0.001
**HbA1c**, mmol/mol	36 (4)	51 (13)	<0.001
**HbA1c**, %	5.4 (0.5)	6.8 (1.2)	
<53 mmol/mol (<7%), %		62.7	
53–63 mmol/mol (7–7.9%), %		22.0	
>64 mmol/mol (>8%), %		15.3	
**C Peptide**^**a**^, nmol/l		1.07 (0.69)	
**HOMA-IR**^**a**^, %	2.23 (3.35)	5.17 (8.76)	<0.001
**HDL**, mmol/l	1.63 (0.49)	1.29 (0.39)	<0.001
**LDL**, mmol/l	3.13 (0.80)	2.18 (0.75)	<0.001
**Triglycerides**, mmol/l	1.01 (0.58)	1.48 (1.01)	<0.001
**C-reactive protein**, nmol/l	3.24 (7.71)	17.14 (43.72)	<0.001
**RAS blockade**, %	0	70.2	<0.01
ACE inhibitor		19.6	
ARB		59.5	
Anti-aldosterone		4.7	
Dual blockage^**b**^		15.4	

Data are presented as means (standard deviation) or percentages, as appropriate. T2DM, type 2 diabetes; Vascular disease, presence of previous cardiovascular, cerebrovascular and/or peripheral disease; RAS, renin-angiotensin system; ARB, angiotensin II receptor blocker; ^a^patients on insulin therapy were excluded from this analysis. ^b^Dual blockage, concomitant use of ACE inhibitors, ARB and/or anti-aldosterone drugs; eGFR, estimated by CKD-EPI formula based on creatinine and cystatin C; FPG, fasting plasma glucose; N/A: not applicable; NS: not statistically significant.

### MMP-10 and TIMP-1 in type 2 diabetes

**Figure 1:**
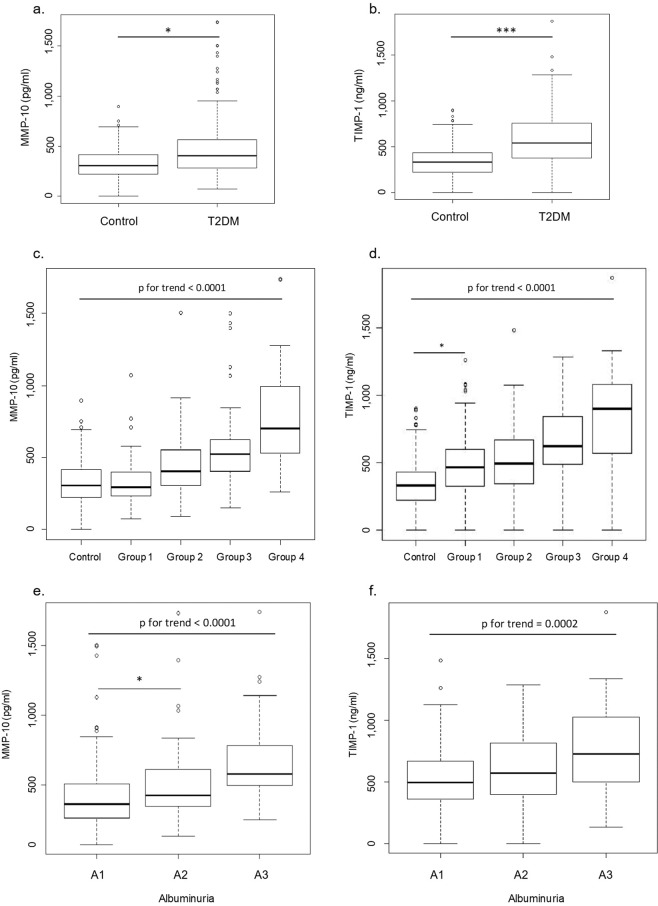
MMP-10 and TIMP-1 serum levels in the studied population. Panels a,b show, respectively, MMP-10 and TIMP-1 serum levels in type 2 diabetes patients (T2DM) and healthy subjects (Control). Comparison of MMP-10 (panel c) and TIMP-1 (panel d) serum levels between healthy subjects (Control) and T2DM patients divided in groups according to CKD stages. Group 1 (n = 89): T2DM with eGFR > 90 ml/min/1.73 m^2^; Group 2 (n = 97): T2DM with eGFR: 90–60 ml/min/1.73 m^2^; Group 3 (n = 54): T2DM with eGFR: 60–30 ml/min/1.73 m^2^; Group 4 (n = 28): T2DM with eGFR < 30 ml/min/1.73 m^2^. MMP-10 and TIMP-1 levels according to albuminuria (panels e, f). Sample spot urinary albumin/creatinine ratio in T2DM patients as A1: < 30 mg/g; A2: 30–300 mg/g; A3: > 300 mg/g. MMP-10 expressed in pg/ml and TIMP-1 in ng/ml. ^*^p < 0.05; ^***^p < 0.001.

**Figure 2:**
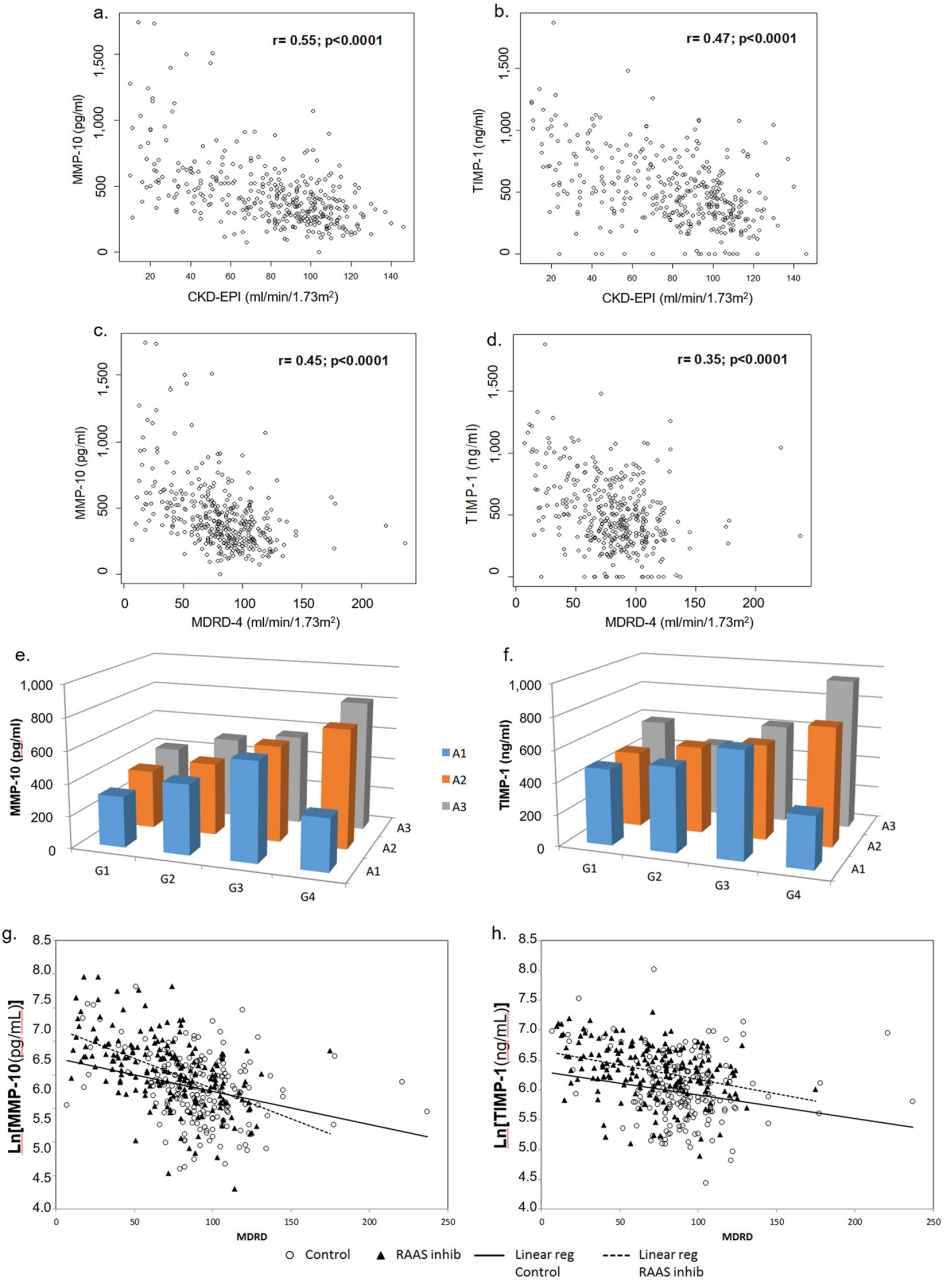
Correlation between MMP-10 and TIMP-1 with renal function. A negative association was found between MMP-10 and TIMP-1 with eGFR using different formulas for GFR estimation, although the strongest correlations were noted with CKD-EPI cystatin C (panels a,b). Crude analysis showed a readily noticeable increase in MMP-10 (panel e) with GFR and albuminuria, which was not as striking for TIMP-1 (panel f). Panels g and h show the association between MMP-10 and renal function (estimated by MDRD) separately in the subgroups of patients taking RAS inhibitors and those not treated. The slope of the regression line was more steep in the group of patients who received RAS inhibitors. Both regression lines crossed at MDRD = 76 ml/min/1.73 m^2^, showing that patients with better renal function treated with RAS inhibitors tend to have lower serum MMP10 concentration. Conversely, patients with worst renal function (MDRD < 76 ml/min/1.73 m^2^) treated with RAS inhibitors tend to have higher serum MMP-10 concentration than non-treated ones. G1 (n = 89): T2DM with eGFR > 90 ml/min/1.73 m^2^; G2 (n = 97): T2DM with eGFR: 90–60 ml/min/1.73 m^2^; G3 (n = 54): T2DM with eGFR: 60–30 ml/min/1.73 m^2^; G4 (n = 28): T2DM with eGFR < 30 ml/min/1.73 m^2^. Albuminuria in T2DM as A1: < 30 mg/g; A2: 30–300 mg/g; A3: > 300 mg/g.

MMP-10 and TIMP-1 were significantly higher (p = 0.02 for MMP-10 and p < 0.001 for TIMP-1) in patients with diabetes compared to controls, after adjusting for age, gender and BMI (Fig. 1a,b). ANCOVA, followed by *post-hoc* comparisons, revealed a steady increase in MMP-10 and TIMP-1 throughout the different stages of CKD in diabetic subjects (p < 0.0001). A positive correlation between MMP-10 and TIMP-1 was noted (R = 0.33, p < 0.0001). Increased levels of MMP-10 and TIMP-1 were found even at early stages of DKD, despite no significant impairment in GFR. TIMP-1 levels elevated earlier than MMP-10 (eGFR > 90 vs. eGFR = 90–60 ml/min/1.73 m^2^) (Fig. 1c,d). Significant differences in MMP-10 and TIMP-1 across stages of albuminuria (p-trend < 0.0001 for MMP-10 and p-trend = 0.0002 for TIMP-1) were found after ANCOVA analysis (Fig. 1e,f). The negative association between MMP-10 and TIMP-1 with eGFR was maintained while using different formulas for GFR estimation, although the strongest correlations were noted with CKD-EPI cystatin C (Fig. 2a–d).

The univariate analysis showed a readily noticeable increase in MMP-10 with GFR and albuminuria (Fig. 2e), which was not as striking for TIMP-1 (Fig. 2f). This implies that the serum levels of both increase as DKD progresses, a process characterized by macroalbuminuria and a greater deterioration in the filtration rate.

The increase in MMP-10 and TIMP-1 throughout the different stages of CKD remained significant (p < 0.0001 both) in the two way ANCOVA (GFR and albuminuria) after adjusting for age, gender and BMI. This statistical model explained variations in MMP-10 (adjusted R = 0.56) better than in TIMP-1 (adjusted R = 0.42). However, the differences between stages of albuminuria disappeared in this statistical analysis. When performing the ANCOVA analysis, we found an interaction (p < 0.05) between RAS inhibitors treatment and GFR that led us to study the association between MMP-10 and renal function separately in the two subgroups of patients. The slope of the regression line was more steep in the group of patients who received RAS inhibitors, as shown by the coefficients [−0.0090 (−0.0111, −0.0069), p = 1.5 10^−14^ vs −0.0053 (−0.0080, −0.0026), p = 1.94 10^−4^]. These two regression lines cross at MDRD = 76 ml/min/1.73 m^2^, so that patients with better renal function treated with RAS inhibitors tend to have lower serum MMP-10 concentration. Conversely, patients with worst renal function (MDRD < 76) treated with RAS inhibitors tend to have higher serum MMP-10 concentration than non-treated ones. However, no interaction was found and no differences were detected when comparing circulating levels of TIMP-1 (Fig. 2g,h) (see Supplementary Fig. S2). However, the magnitude of this effect was not big enough to find statistically significant differences when comparing circulating levels of MMP-10 between patients on RAS treatment and patients without RAS inhibitors, despite comparing groups with similar renal function (see Supplementary Fig. S2). No interaction was found and no differences were detected when comparing circulating levels of TIMP-1 (Fig. 2h; see Supplementary Fig. S2).

### Experimental study

A study was conducted in db/db mice to explore whether renal expression of *Mmp10* and *Timp1* may be up-regulated in this diabetic model, recapitulating our observations in the clinical setting. Moreover, since prior data has supported a link between RAS and other MMPs, we evaluated if renal *Mmp10* and *Timp1* expression could be modulated through RAS blockade with telmisartan. At 8 weeks of age, all db/db mice became obese (40.1 ± 2.7 vs. 24.9 ± 1.3 g; p < 0.001) and developed T2DM, demonstrated by hyperglycaemia and hyperinsulinemia (Table 2). Following the onset of diabetes, there was a progressive increase in albuminuria between 8 and 16 weeks of age, which was not observed in controls. After 8 weeks of treatment, those diabetic animals treated with telmisartan displayed a significant reduction in albuminuria in comparison with non-treated diabetic mice. Diabetic mice at 16 weeks of age presented higher plasma creatinine than controls, but no changes were observed between db/db mice treated with telmisartan and those without treatment (Table 2). No changes were observed with blood pressure either (db/db + T: 123.5 ±11.3 vs db/db: 127.7 ±15.5 mmHg, p = 0.23).

**Table 2:** Characteristics of db/db (DM) and db/m (Control) mice, treated (+T) and untreated with telmisartan.

8 weeks		Control		DM		p
Weight, g		24.9 (1.3)		40.1 (2.7)		<0.001
Glucose, mmol/l		13.15 (3.22)		24.75 (7.94)		<0.01
Insulin, pmol/l		368.8 (231.3)		2446.7 (415.3)		<0.001
Creatinine, μmol/l		12.38 (5.30)		13.26 (6.19)		NS
uACR, mg/mmol		2.23 (0.09)		14.16 (4.03)		0.01
**12 weeks**	**Control**	**DM**	**p**	**Control + T**	**DM + T**	**p**
Weight, g	27.9 (1.7)	43.3 (3.5)	<0.001	28.1 (1.4)	45.6 (3.0)	<0.001
Glucose, mmol/l	15.93 (3.44)	48.62 (8.05)	<0.001	16.59 (7.49)	41.85 (10.88)	<0.001
Insulin, pmol/l	528.5 (360.5)	1359.8 (500.7)	<0.001	492.4 (286.8)	1433.5 (433.4)	<0.001
Creatinine, μmol/l	17.68 (0.00)	28.29 (7.07)	0.01	21.22 (5.30)	25.64 (7.07)	NS^a^
uACR, mg/mmol	1.64 (1.28)	16.06 (5.39)	0.01	0.92 (0.48)	10.36 (3.81)	NS
**16 weeks**	**Control**	**DM**	**p**	**Control + T**	**DM + T**	**p**
Weight, g	28.2 (1.3)	48.9 (3.0)	<0.001	28.9 (2.2)	43.6^b^ (4.7)	<0.001
Glucose, mmol/l	10.38 (2.39)	36.13 (7.21)	<0.001	14.37 (3.05)	45.12 (6.55)	<0.001
Insulin, pmol/l	118.8 (75.7)	1518.9 (950.8)	<0.001	184.0 (110.4)	811.9^c^ (386.1)	0.03
Creatinine, μmol/l	7.07 (2.65)	30.06 (32.71)	0.01	13.26 (7.07)	24.75 (3.54)	NS^a^
uACR, mg/mmol	1.28 (1.71)	33.12 (27.48)	<0.001	1.88 (1.72)	17.68^d^ (9.08)	0.01

Control: db/m mice; DM: db/db mice; Control +T: db/m + telmisartan mice; DM + T: db/db + telmisartan mice. Data shown are mean ± SD. *p* values adjusted by Bonferroni; ^a^DM + T vs. DM ^b^p < 0.0001 vs. DM; ^c^p < 0.01 vs. DM; ^d^p < 0.05 vs. DM; NS: not statistically significant.

**Figure 3:**
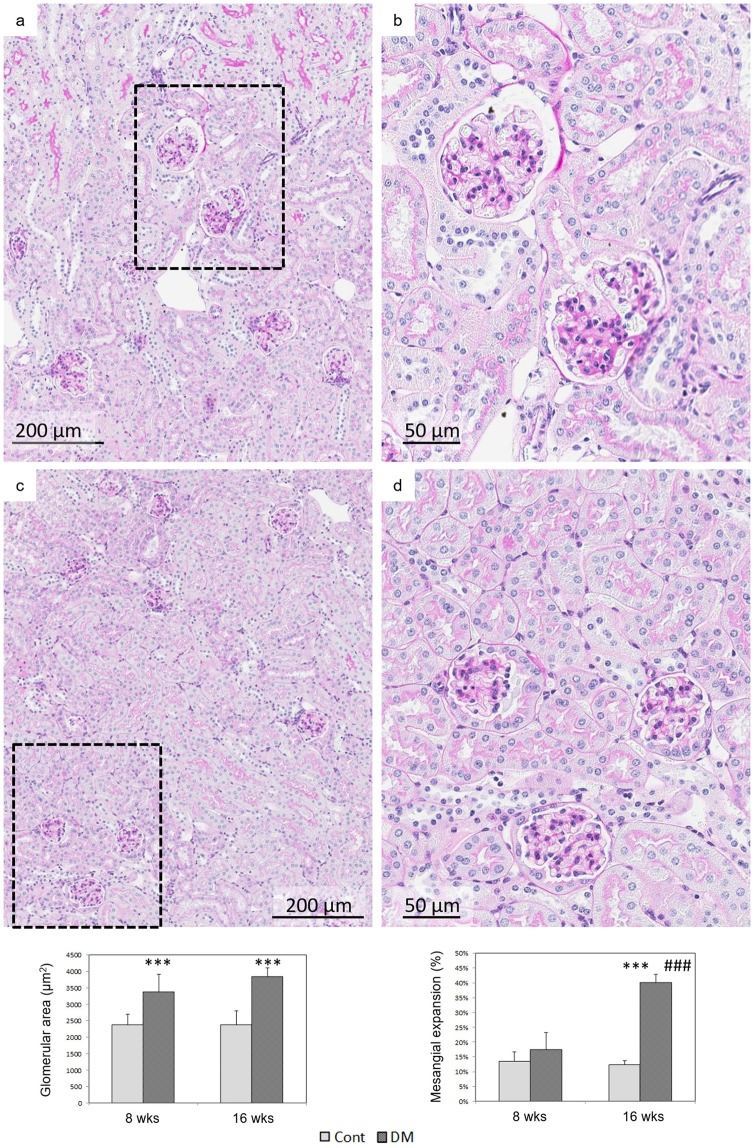
Histological changes in 16 weeks db/db and db/m mice. Periodic acid Schiff stains in 16 weeks db/db (DM) (panels a,b) and db/m (Cont) mice (panels c, d). A greater mesangial matrix expansion was observed in 16 weeks db/db mice compared to age-matched db/m and db/db of 8 weeks-old. Glomerular hypertrophy was observed in db/db (8 and 16 weeks) compared to age-matched db/m (graphics). ^***^p < 0.001 vs. db/m; ^###^p < 0.001 vs. 8 weeks-old db/db. n = 6 on each group.

At 16 weeks of age, glomerular hypertrophy and mesangial matrix expansion were significantly increased in db/db mice (Fig. 3a–d). In spite of the noticeable glomerular hypertrophy, the mesangial matrix expansion was not significantly different in db/db compared with db/m mice at age 8 weeks (Fig. 3). Altogether, these results confirmed the suitability of the model to analyse early stage of DKD changes at 8 weeks and mild changes at 16 weeks.

### Renal expression of *Mmp10* and *Timp1* and their modulation by telmisartan

**Figure 4:**
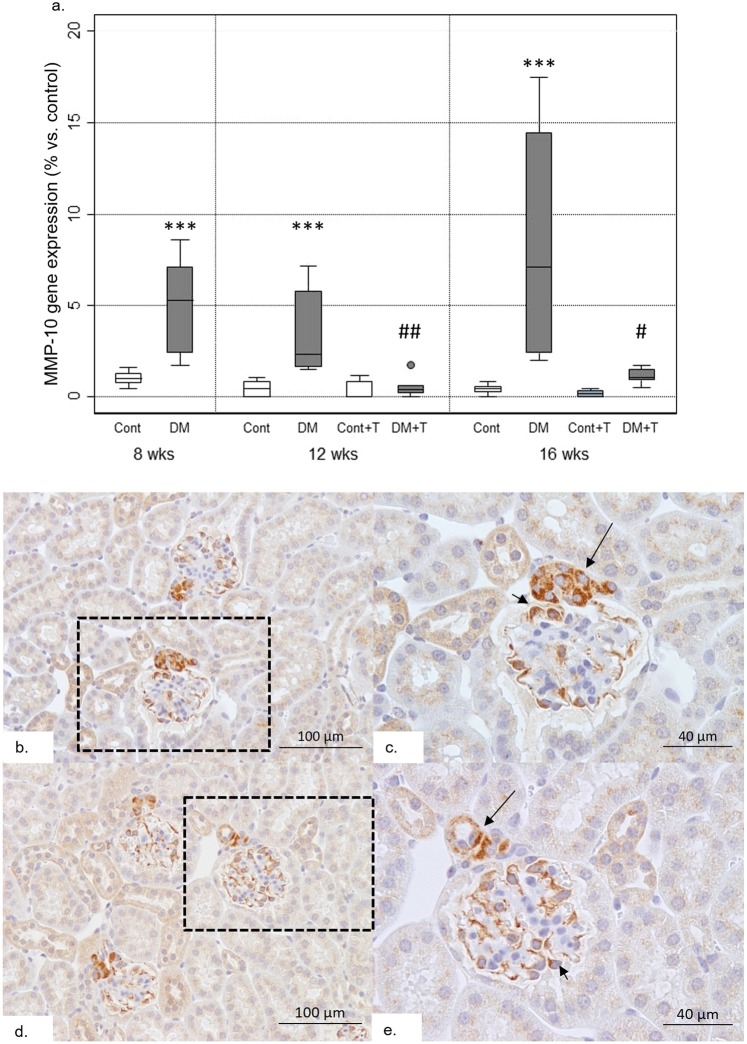
Renal MMP-10 gene expression. Panel a shows a greater renal *Mmp10* expression in db/db (DM) vs. db/m (Cont). Treatment with telmisartan (+T) resulted in down-regulation of *Mmp10* expression. Renal MMP10 immunostaining (panels b–e) has been found to be mainly located in podocytes (short arrows) and juxtaglomerular apparatus (long arrows) of db/db mice. DM: db/db; Cont: db/m; DM + T: db/db treated with telmisartan; Control + T: db/m treated with telmisartan. ^***^p < 0.001 vs. db/m; ^##^p < 0.01 vs. 12 weeks-old db/db without telmisartan; ^#^p < 0.05 vs. 16 weeks-old db/db without telmisartan. n = 6 on each group.

At 8, 12 and 16 weeks-old, db/db mice showed increased renal gene expression of *Mmp10* compared to age and sex-matched db/m mice. The highest *Mmp10* gene expression was observed at 16 weeks of age, with an 8-fold increase versus controls (Fig. 4a). A non-significant increment on *Timp1* gene expression was noted in diabetic mice (p = 0.21), as well as a down-regulation after RAS inhibition which did not achieve statistical significance (see Supplementary Fig. S3). Immunohistochemical analysis was performed to evaluate the localization of MMP-10 expression in the kidney. Positive staining was observed in podocytes and juxtaglomerular apparatus from diabetic mice (Fig. 4b–e). No specific staining was observed in tubules or interstitium. Treatment with telmisartan significantly downregulated *Mmp10* gene expression after 4 (p < 0.01) and 8 weeks (p < 0.05), to nearly the normal expression in control mice (Fig. 4a). A significant positive correlation was observed in diabetic mice between renal *Mmp10* gene expression and albuminuria (R = 0.792; p = 0.019).

## Discussion

Our study demonstrates that circulating MMP-10 is increased in T2DM, together with its inhibitor TIMP-1, starting at the earliest stages of DKD, and their concentration increases with the severity of kidney disease. Moreover, we demonstrated overexpression of renal *Mmp10* in an experimental murine model of T2DM, which was prevented by RAS inhibition.

Increased serum levels of MMP-10 and TIMP-1 were found in patients with diabetes compared to healthy subjects. The increment was observed in those with glomerular filtration rates greater than 60 and 90 ml/min/1.73 m^2^ for MMP-10 and TIMP-1, respectively. In addition, a progressive elevation occurred across different stages of CKD with the highest values in patients with advanced kidney disease. A strong correlation was demonstrated between both proteins and glomerular filtration rate estimated by traditional equations, especially with cystatin C in comparison with serum creatinine. An experimental murine model was carried out, with the aim of studying the renal expression of *Mmp10* and *Timp1* during hyperglycaemia. We were able to demonstrate glomerular overexpression of *Mmp10* in diabetic mice from the earliest stages of DKD. *Mmp10* downregulation occurred after RAS blockade with telmisartan during 4 and 8 weeks of treatment, indicating an involvement of the RAS system.

The role of other MMPs in DKD has been previously studied^2,12–14^. Two recent publications have demonstrated association between MMP-10 and cardiovascular risk factors in T1DM^4,15^. Consistent with our results, Toni *et al*. observed higher MMP-10 levels in T1DM compared to controls^4^, however information has been lacking about the association between MMP-10 and T2DM. We also saw an increase in TIMP-1 levels in the population with diabetes. Several studies confirm our results in which the elevation of TIMP-1 is demonstrated in patients with DM both in the presence or absence of overt vascular complications^5,15^. A few studies have shown divergent results compared to ours but had either a different study population (mean age: 19 years-old)^2^ or a low sample size^7^. The observed correlation between serum MMP-10 and TIMP-1 in DM reflects the coordinated up-regulation of MMPs and TIMPs that has been reported in other studies^16^.

We further evaluated MMP-10 and TIMP-1 according to renal function as recent studies have demonstrated that DKD can exist despite the absence of biochemical indicators^17–19^, and elevation of other MMPs may precede the development of microalbuminuria^7^. A parallel increase was observed between both proteins in relation to the progression of renal disease. Of note is the increase in MMP-10 and TIMP-1 in T2DM despite the absence of a significant decrease in eGFR: TIMP-1 begins to increase when the eGFR is still above 90 ml/min/1.73 m^2^, while MMP-10 increases with an eGFR lower than 90, but greater than 60 ml/min/1.73 m^2^. Previous data has shown an imbalance between other MMPs and TIMP-1 in DM, even in the absence of overt nephropathy^7,20^, however this is the first study showing an elevation of serum MMP-10 in early-stage DKD, followed by a progressive increase towards late-stage disease. In addition, an inverse correlation was observed between MMP-10 and TIMP-1 with eGFR, in line with previous results from our group that described higher levels of MMP-10 and TIMP-1 in patients with advanced CKD^5^. The previous study estimated GFR by MDRD-4, while in the present work we evaluated four different formulas (MDRD-4, CKD-EPI creatinine, CKD-EPI cystatin C, CKD-EPI creatinine-cystatin C), showing that the strongest correlation is that based on cystatin C.

Despite not being a *sine qua non* condition, the presence of albuminuria is one of the characteristic signs of DKD. In our study, we found an association between the level of albuminuria and higher circulating levels of MMP-10 and TIMP-1. However, this association disappears after adjusting for GFR, but was not modified after adjusting for RAS treatment. The reason of this may be due to the fact that the level of albuminuria may not be fully dependent on the degree of GFR reduction (i.e. patients with low GFR may have either high or low uACR). On the other hand, in our cohort the patients using RAS inhibitors had a greater albuminuria compared to those without treatment, which may explain the variability in MMP-10 levels in this population. The relationship between albuminuria and MMP-10 has also been described by Peeters *et al*. who observed a significant association between the degree of albuminuria and circulating MMP-10 and TIMP-1 in patients with T1DM^15^. This association is interesting as their study population had a mean eGFR of 95.7 ml/min/1.73 m^2^ and the association held good for normo-, micro- and macroalbuminuric patients.

To assess the role of renal tissue as a possible source of MMP-10 and TIMP-1 overexpression, and coupled to data showing an association between other MMPs and RAS^12,21,22^, we analysed a murine model to test whether the renal expression of both proteins could be up-regulated during hyperglycaemic state and if their expression could be downregulated with telmisartan. Higher *Mmp10* renal expression was observed from early stage of DKD in db/db as compared to db/m, even in the absence of significant histological lesions (8 weeks-old), and a down-regulation was observed after RAS blockade. Moreover, a correlation between glomerular *Mmp10* expression and albuminuria was demonstrated. Immunohistochemical analysis showed MMP-10 localization in podocytes and juxtaglomerular apparatus known to have high RAS activity^23^. These findings may support the relationship between MMP-10 and RAS, and a pathogenic role of MMP-10 in glomerular DKD. In agreement with data from other studies, reporting increased glomerular *Timp1* expression in diabetic rats^24^, we also observed higher *Timp1* expression in renal tissue in the diabetic mice, however our results did not achieve statistical significance probably due to the limited sample size.

The role of MMP-10 in kidney disease has not been extensively studied. Toni *et al*. demonstrated that diabetic mice with *Mmp10* deficiency showed minor renal morphologic alterations, as well as improved renal function, compared to those expressing *Mmp10*^4^. Keeping this in mind, our results would suggest a deleterious glomerular effect for MMP-10 in DKD and show that its expression can be down-regulated by blocking RAS. The mechanism involving MMP-10 in the pathophysiology of DKD is not yet clear. Two recent studies have analysed MMP-10 in primary glomerular disease^25,26^. The authors demonstrated the presence of MMP-10 in podocytes using immunofluorescence and suggest a regulation of the metalloproteinase through focal adhesion kinase (FAK) activation. Since FAK activation is at the beginning of the angiotensin II signalling pathway^27,28^, we propose a possible link between MMP-10 and RAS in DKD, where angiotensin II, induced by hyperglycaemia, could stimulate FAK activation resulting in *Mmp10* overexpression. In this scenario, the significant down-regulation of renal *Mmp10* expression achieved following RAS blockade by telmisartan would be explained. However, this hypothesis must be demonstrated by complementary mechanistic studies. In our experimental model, telmisartan dosage was chosen so that RAS inhibition did not produce changes in blood pressure, indicating that reduction in *Mmp10* expression was independent of hemodynamic factors other than DKD.

Certain limitations of our study must be acknowledged. The clinical study is a cross-sectional observational study and prospective long-term studies are needed to stablish a causal link between MMP-10 and TIMP-1 dysregulation and the onset of DKD. Prospective long-term studies are also necessary to identify whether MMP-10 and TIMP-1 could be used as diagnostic and prognostic biomarkers. Another limitation is that we did not reach enough statistical power to detect statistically significant differences between patients with or without RAS inhibitors, with similar GFR, even when we compared GFR > 90 or GFR < 60 ml/min/1.73 m^2^. In addition, confounding factors such as markers of chronic inflammation or subclinical atherosclerosis were not analysed as they did not form part of the original objectives for the study. In the experimental model, an important limitation is the difficulty in measuring the concentration of circulating MMP-10 in mice, as we have found no ELISA kits with the required specificity (especially, differentiating MMP-10 from MMP-3), sensitivity and reliability. Information is lacking about urinary concentration of both proteins, as well as a comparison of the results with other glomerular diseases.

Interestingly, the findings of the present study are relevant for identifying an up-regulation of MMP-10 and TIMP-1 in T2DM, even before overt kidney disease is noted, and opens the door for future studies aiming at elucidating the mechanistic role of this MMP and its inhibitor on DKD.

In conclusion, we demonstrate for the first time an association between MMP-10 and TIMP-1 and the different stages of renal disease, as well as glomerular overexpression of *Mmp10* in T2DM, which is prevented by blocking RAS. The novelty of these results lies in the participation of MMP-10 in diabetic glomerular disease, as well as its possible role as a new therapeutic target.

## Supplementary information

Supplementary data.

## Data Availability

The datasets generated during and/or analysed during the current study are available from the corresponding author on reasonable request.
