# Supplementary material for: MMP-10 is Increased in Early Stage Diabetic Kidney Disease and can be Reduced by Renin-Angiotensin System Blockade
Source: Sci Rep. 2020 Jan 8;10:26. doi: 10.1038/s41598-019-56856-3 (PMC6949265; doi:10.1038/s41598-019-56856-3)
Supplement: Supplementary file 1 — Supplementary data. [file 41598_2019_56856_MOESM1_ESM.pdf]

Supplementary data:

MMP-10 IS INCREASED IN EARLY STAGE DIABETIC KIDNEY DISEASE AND CAN BE REDUCED BY RENIN-ANGIOTENSIN SYSTEM BLOCKADE

José María Mora-Gutiérrez · José Antonio Rodríguez · María A. Fernández-Seara · Josune Orbe · Francisco Javier Escalada · María José Soler · María Fernanda Slon-Roblero · Marta Riera · José Antonio Páramo · Nuria Garcia-Fernandez*

**Corresponding author:* Garcia-Fernandez, Nuria. Orcid ID: 0000-0003-1082-0806

Clínica Universidad de Navarra. Avenida Pio XII, 36. Pamplona 31008, Navarra, Spain.

Avenida Pio XII, 36. Pamplona 31008, Navarra, Spain. Email: nrgarcia@unav.es

Figures

Supplementary Figure S1 Flowchart of study recruitment

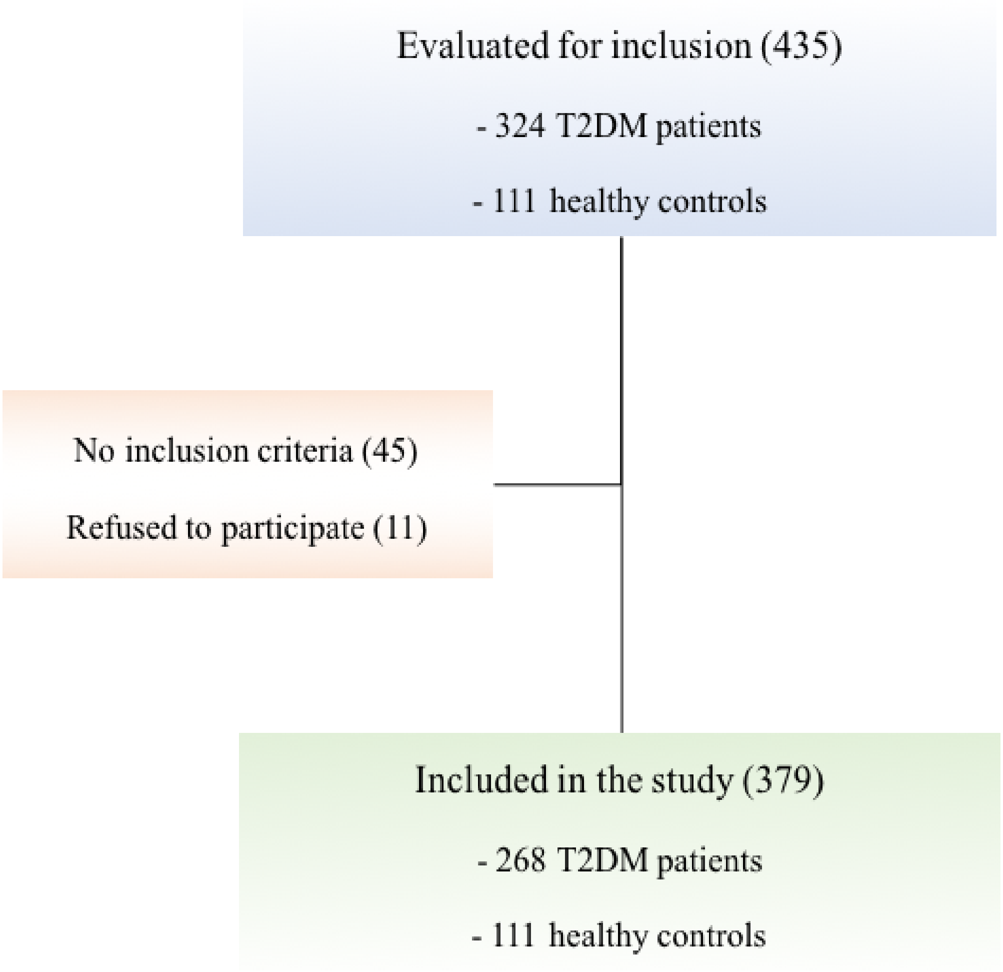

Supplementary Figure S1 A total of 324 consecutive type 2 diabetes (T2DM) patients and 111 healthy volunteers were recruited over a period of 24 months. Of these diabetic subjects, 11 declined participation in the study and 45 patients did not fulfill inclusion criteria. From the total recruited, 379 subjects were finally studied (268 T2DM patients and 111 healthy controls)

Supplementary Figure S2 MMP-10 and TIMP-1 serum levels in type 2 diabetic patients corresponding to group 1

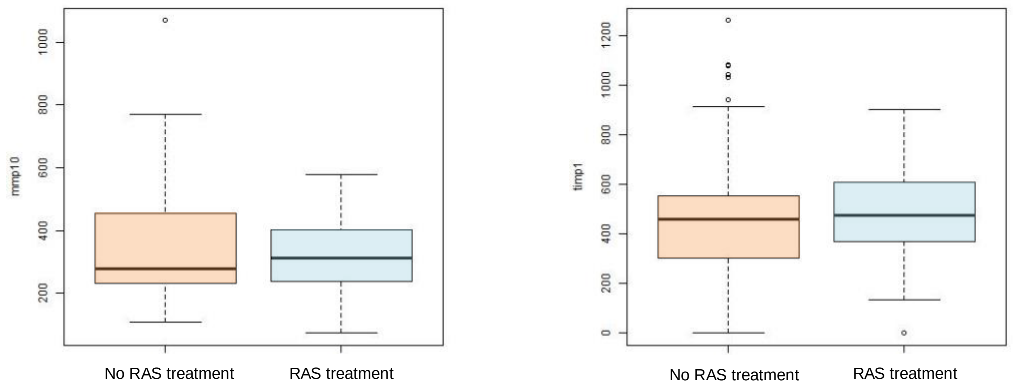

MMP-10 [pg/ml]			
No RAS		RAS	
Mean	SD	Mean	SD
351	191	321	122
Median	IQR	Median	IQR
278	224	313	157

TIMP-1 [ng/ml]			
No RAS		RAS	
Mean	SD	Mean	SD
503	301	483	222
Median	IQR	Median	IQR
459	253	476	236

Supplementary Figure S2 MMP-10 and TIMP-1 serum levels in type 2 diabetic patients with similar renal function. The graphic and table correspond to group 1 subjects (n=89): T2DM with eGFR >90ml/min/1.73m^2

Supplementary Figure S3 Renal TIMP-1 gene expression

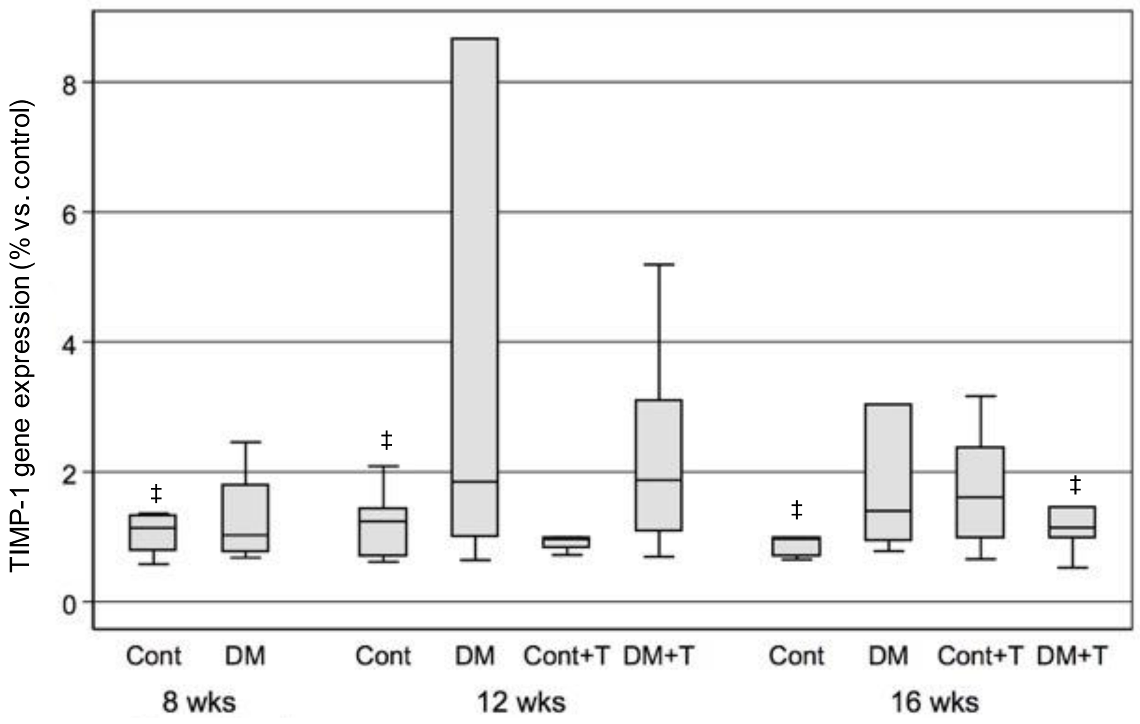

Supplementary Figure S3 Renal expression of *Timp1* was moderately higher in db/db (DM) compared to db/m (Cont) and a down-regulation was observed after RAS inhibition, but these differences did not achieve statistical significance. DM: db/db; Cont: db/m; DM+T: db/db treated with telmisartan; Control+T: db/m treated with telmisartan. ‡p>0.05 vs. same-aged db/db mice. n=6 on each group
